# Partial root-zone drying subsurface drip irrigation increased the alfalfa quality yield but decreased the alfalfa quality content

**DOI:** 10.3389/fpls.2024.1297468

**Published:** 2024-02-06

**Authors:** Yadong Wang, Chong Xu, Qian Gu, Yalong Shi, Jiale Chen, Honghui Wu, Jing He, Xingfu Li, Liliang Han, Derong Su

**Affiliations:** ^1^State Key Laboratory of Efficient Utilization of Arid and Semi-arid Arable Land in Northern China, Chinese Academy of Agricultural Sciences, Beijing, China; ^2^College of Grassland Science, Beijing Forestry University, Beijing, China; ^3^Industry Development and Planning Institute, National Forestry and Grassland Administration of P.R. China, Beijing, China; ^4^Academy of Forestry Inventory and Planning, National Forestry and Grassland Administration of P.R. China, Beijing, China

**Keywords:** lucerne, nutritional characteristics, partial root-zone drying, regulated deficit irrigation, subsurface drip irrigation

## Abstract

Water shortage seriously restricts the development of grassland agriculture in arid land and dramatically impacts alfalfa (*Medicago sativa* L.) quality content and hay yield. Reasonable irrigation methods have the potential to enhance the alfalfa quality content, hay yield, and thus quality yield. Whether partial root-zone drying subsurface drip irrigation (PRDSDI) improves the alfalfa quality yield, quality content, and hay yield is still unknown compared with conventional subsurface drip irrigation (CSDI). The effects of PRDSDI compared with that of CSDI and the interaction with irrigation volume (10 mm/week, 20 mm/week, and 30 mm/week) on the alfalfa quality yield were investigated in 2017–2018 and explained the change in quality yield with the alfalfa quality content and hay yield. Here, the results showed that PRDSDI did not increase the alfalfa quality yield in 2 years. PRDSDI significantly increased acid detergent fiber by 13.3% and 12.2% in 2018 with 10-mm and 20-mm irrigation volumes and neutral detergent fiber by 16.2%, 13.2%, and 12.6% in 2017 with 10-mm, 20-mm, and 30-mm irrigation volumes, respectively. PRDSDI significantly decreased the crude protein by 5.4% and 8.4% in 2018 with 10-mm and 20-mm irrigation volumes and relative feed value by 15.0% with 20-mm irrigation volume in 2017 and 9.8% with 10-mm irrigation volume in 2018, respectively. In addition, PRDSDI significantly increased the alfalfa average hay yield by 49.5% and 59.6% with 10-mm and 20-mm irrigation volumes in 2018, respectively. Our results provide a counterexample for PRDSDI to improve crop quality. Although there was no significant improvement in average quality yield by PRDSDI, the positive impact of average hay yield on quality yield outweighed the negative impact of quality content. Thus, it has the potential to improve quality yields. The novel findings regarding the effects of PRDSDI on quality yield are potentially favorable for the forage feed value in water-limited areas.

## 1 Introduction

Alfalfa (*Medicago sativa* L.) is a critical element in dairy cow diets. Because of insufficient domestic production in China, there has been a steady increase in imports from abroad over the past decade (Wang and Zou, 2020; Wang and Zhang, 2023). Despite the fact that 90% of the alfalfa planting area is in Northern China, where the alfalfa produced is of good quality and high yield (Feng et al., 2022; Kamran et al., 2022), water shortage remains a key limiting factor for alfalfa production (Wang et al., 2021; Chen et al., 2023). To address this challenge and enhance the alfalfa quality and hay yield, various water-saving irrigation methods have been implemented (Li et al., 2023; Liu et al., 2023).

The issue of water shortage for irrigation is becoming increasingly serious worldwide, particularly in the developing countries (Schmitt et al., 2022). It is imperative to establish a proper framework for the irrigation techniques tailored to specific crops and purposes, whether the crop is grown for forage, grains, or fiber (Chen et al., 2020). It is very necessary to manage properly the irrigation water for crop production and forage quality (Jiang et al., 2022; Xu et al., 2022). Developing proper water use strategy for the vegetation and forage production, especially under the deficit irrigation (Li et al., 2018; El Mouttaqi et al., 2023), is essential to improve the forage quality and ensure sustainable vegetation production (Fu et al., 2022). Numerous water-saving irrigation methods have been devised for the vegetation and forage production, including furrow irrigation, sprinkler irrigation, and subsurface drip irrigation (Li et al., 2023; Wu et al., 2023). Among them, subsurface drip irrigation is more water-saving, as it replenishes water directly in the water-absorbing zone of plants’ roots, in contrast to furrow irrigation and sprinkler irrigation that add water to the soil surface (Hutmacher et al., 2001; Liu et al., 2021a). However, conventional subsurface drip irrigation (CSDI) often involves large lines spacing, resulting in uneven irrigation where plants closer to the scuppers receive more water than those farther away (Lamm, 2012). The typical solution is to increase the irrigation volume to reach plants farther from the scuppers (Hutmacher et al., 2001; Kandelous et al., 2012). To address the issue of uneven irrigation in CSDI, another irrigation method, namely, partial root-zone drying subsurface drip irrigation (PRDSDI), has been recently developed (Wang et al., 2021). PRDSDI can be viewed as a doubled CSDI, involving the addition of a second subsurface drip irrigation system on the base of CSDI. The scuppers of the second system are located in the middle of the scuppers of the first system, allowing for more even irrigation of plants with the same irrigation volume (Wang et al., 2021). Thus, using the same irrigation volume, CSDI can irrigate plants more evenly than PRDSDI by alternating the two subsurface drip irrigation systems between irrigation events (Wang et al., 2021).

More evened irrigation provides a stable soil water environment for plants (Wang et al., 2021). However, PRDSDI has not consistently demonstrated a promotion in plant yield (Jovanovic et al., 2010; Çolak et al., 2018) and, in some cases, has even reduced hay yield (Wang et al., 2021). The pleasant surprise is that the PRDSDI showed the advantage of improving plant quality content compared with CSDI (Jovanovic et al., 2010; Çolak et al., 2018). Nevertheless, in previous research comparing CSDI under full irrigation, PRDSDI did not show the advantage of improving quality content (Wang et al., 2021). It remains uncertain whether PRDSDI improves alfalfa hay yield under varying irrigation volumes, particularly under deficit irrigation, and whether there is an improvement in quality content.

Maintaining a balance between quality content and yield is crucial, particularly for alfalfa, a forage that is valued for its quality content (McCullock et al., 2014; Peake et al., 2019). Focusing solely on achieving high hay yield while undervaluing the alfalfa quality content may diminish its market competitiveness (Zhang et al., 2020; Wang et al., 2023). Therefore, investigating the synergistic regulation mechanism between the alfalfa quality content and the hay yield is essential for enhancing its overall quality and production efficiency. In this context, we introduce the concept of the alfalfa quality yield to assess the relationship between the alfalfa quality content and the hay yield (Zhang et al., 2018; Deng et al., 2020). Existing studies on quality yield have primarily centered on biomass-based vegetables (Bumgarner et al., 2012; Nyathi et al., 2019), pastures (Zhang et al., 2018; Deng et al., 2020), and other crops (Graham et al., 2018; Li et al., 2021).

This study aims to compare the effects of irrigation methods and the interaction with irrigation volumes on the alfalfa quality yield, alfalfa quality content, and hay yield. We hypothesize that PRDSDI might enhance the alfalfa quality content and hay yield and thus increase its quality yield compared with CSDI. The goal is to explore whether PRDSDI offers advantages in improving the alfalfa quality yield to address the challenge of water limitation constraining the sustainable development of grassland agriculture in arid areas.

## 2 Materials and methods

### 2.1 Site description and experimental design

**Figure 1:**
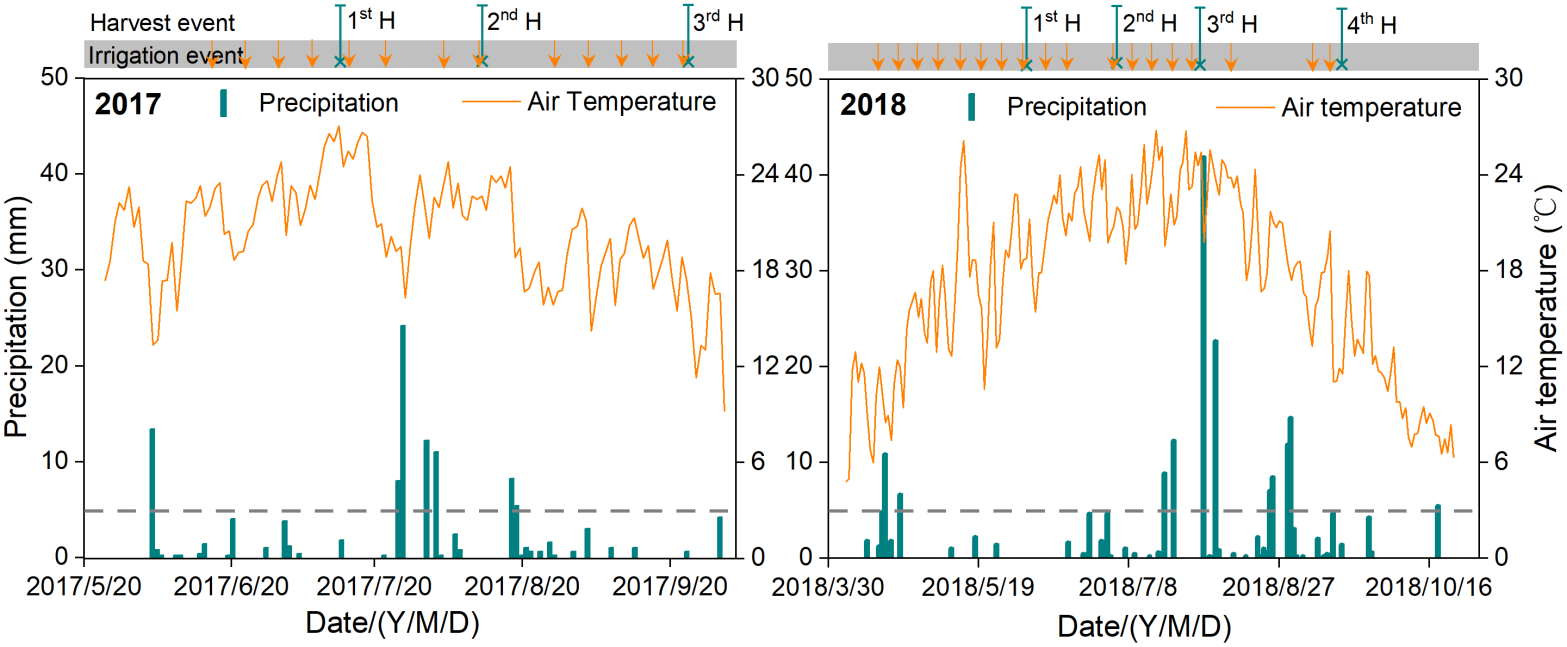
Harvest event, irrigation event, precipitation, and air temperature in the experimental years. The orange arrow indicates the irrigation event time (seedling irrigation not included), and the green × line indicates the harvest event time. (For interpretation of the references to color in this figure legend, the reader is referred to the web version of this article).

The experiments were carried out at the National Field Scientific Observation and Research Station of Oasis Agroecosystem (35°52′N and 102°50′E; altitude, 1,581 m), located in northwest arid land China. The mean annual precipitation and pan evaporation in this region are 164 mm and 2,000 mm, respectively. The mean annual temperature is 8.8°C, and the annual accumulated temperature (>0°C) is 3,550°C. The air temperature and daily precipitation during 24 June to 30 September 2017 and 8 May to 20 September 2018 as well as the alfalfa growing seasons are shown in **Figure 1**. The soil of the field is sandy loam, with an average field capacity, wilting point, and soil bulk density of 0.29 cm^3^ cm^−3^, 0.09 cm^3^ cm^−3^, and 1.50 g cm^−3^ in the upper 1.6 m of soil, respectively.

The irrigation methods included CSDI and PRDSDI. Three irrigation volumes were set for the comparison of PRDSDI and CSDI. Six treatments had 18 subplots in total, and the experimental subplots were arranged in split area. The irrigation volume of 30 mm (CSDI3 and PRDSDI3) was used to represent the full irrigation of alfalfa consumption by local farmers (Kou, 2014). The other two irrigation volumes used full irrigation water for 2/3 (CSDI2 and PRDSDI2) and 1/3 (CSDI1 and PRDSDI1) (**Table 1**), respectively. On 29 July 2017, 19 August, 2017, 8 August 2018, 19 August 2018, 26 August 2018, and 2 September 2018, the irrigation schedule was delayed by 1 week because of excessive precipitation (**Figure 1**). The irrigation frequency was 13 times in 2017 and 19 times in 2018 (**Table 1**; **Figure 1**).

**Table 1:** Irrigation regime details in 2017 and 2018.

Treatment^a^	2017				2018			
	Irrigation volumes (mm week^−1^)	Irrigation frequency (no.)	Irrigation quota (mm)	Irrigation date (day-month)	Irrigation volumes (mm week^−1^)	Irrigation frequency (no.)	Irrigation quota (mm)	Irrigation date (day-month)
CSDI1	10	13	130	24 June, 1 July, 8 July, 15 July, 22 July, 5 August, 12 August, 27 August, 3 September, 10 September, 16 September, 23 September	10	19	190	15 April, 22 April, 29 April, 6 May, 13 May, 20 May, 27 May, 4 June, 10 June, 17 June, 24 June, 3 July, 8 July, 15 July, 22 July, 29 July, 12 August, 1 September, 17 September
PRDSDI1	10	13	130		10	19	190	
CSDI2	20	13	260		20	19	380	
PRDSDI2	20	13	260		20	19	380	
CSDI3	30	13	390		30	19	570	
PRDSDI3	30	13	390		30	19	570	

a PRDSDI represents partial root-zone drying subsurface drip irrigation; CSDI represents conventional subsurface drip irrigation.

**Figure 2:**
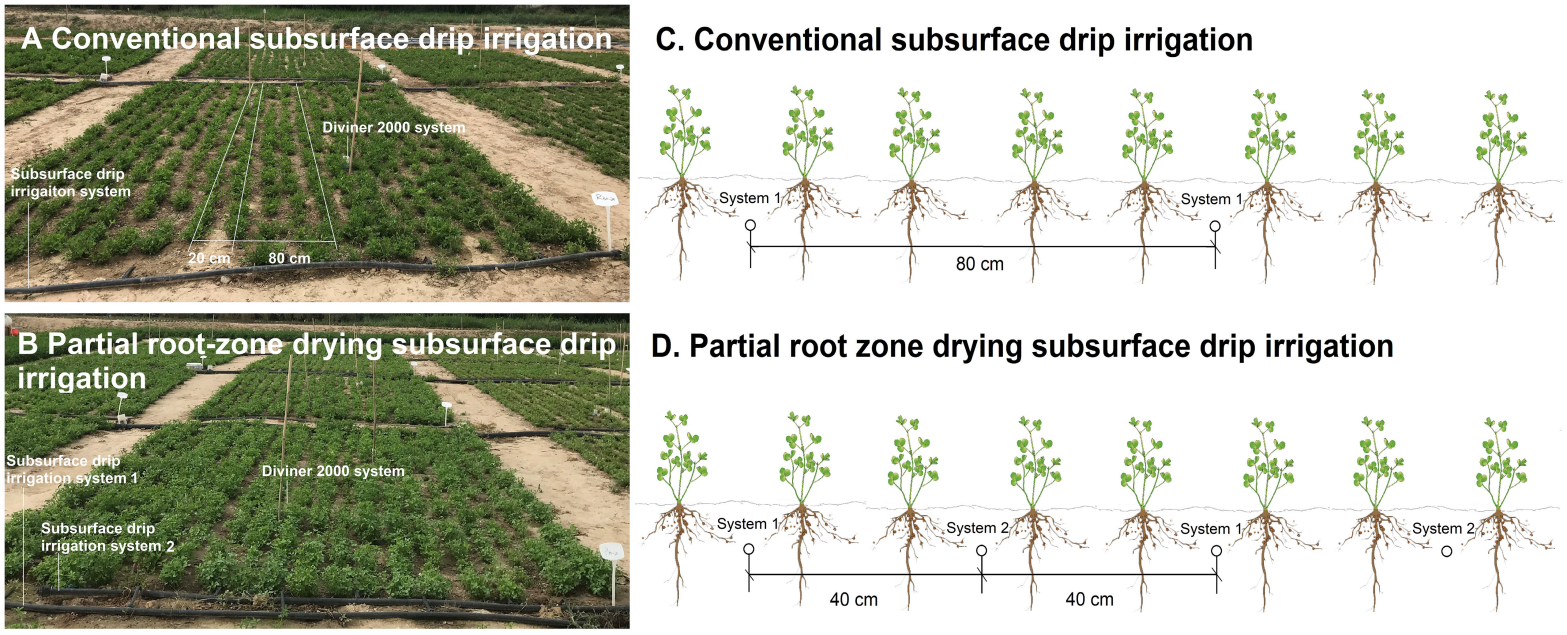
Photograph and layout of the proposed subsurface drip irrigation systems for conventional subsurface drip irrigation **(A, C)** and partial root-zone drying subsurface drip irrigation **(B, D)**. Partial root-zone drying subsurface drip irrigation water supply adopts two subsurface drip irrigation systems, subsurface drip irrigation system 1 provides water during one irrigation event, whereas subsurface drip irrigation system 2 supplies water during the next irrigation event (for interpretation of the references to color in this figure legend, the reader is referred to the web version of this article).

The subsurface drip irrigation system (DAYU Water-saving Group Co. Ltd., Gansu, China) was buried at the depth of 0.2 m before the alfalfa was established in the plots, with a discharge rate of 3 L h^−1^ and 0.3-m interval inline emitters. As we presented earlier, two sets of subsurface drip irrigation systems were arranged to establish PRDSDI (Wang et al., 2021). Thus, the subsurface drip irrigation system 1 is used for 1, 3, 5, ... irrigation events and subsurface drip irrigation system 2 is used for 2, 4, 6, ... irrigation events (**Figure 2**).

On 20 May 2017, Alfalfa (cv. MF4020) was planted with a drill at a rate of 20 kg ha^−1^. The sowing depth was 0.05 m, and the row space was 0.2 m. Pest control was applied as per best management practices throughout the experiment, and weeds were removed manually after each harvest.

### 2.2 Sampling and measurements

The plots were harvested three times during the growing season in 2017 (15 July, 18 August, and 30 September) and four times in 2018 (7 June, 5 July, 7 August, and 20 September) (**Table 2**). The alfalfa hay yield was determined by combining a large plot (1 m × 1 m) with a small plot (0.2 m × 0.2 m). The fresh grass yield was determined after harvest by randomly selecting five samples in the test area and weighing them. The alfalfa moisture content was measured in the small plot, and the hay yield of the 1 m × 1 m sample plot was calculated on the basis of fresh weight with a large sample plot. The samples from the small plot were placed in an oven for 1 h at 105°C for sterilization. The temperature was then lowered to 65°C, where it was maintained until the weight stabilized. Average hay yield was calculated as the average of three harvests in 2017 and the average of four harvests in 2018.

**Table 2:** Harvest regime details in 2017 and 2018.

Regrowth cycle	2017			2018		
	Start date	Harvest date	Regrowth duration (days)	Start date	Harvest date	Regrowth duration (days)
First harvest	20 May^a^	15 July	58	8 April	7 June	62
Second harvest	16 July	18 August	35	8 June	5 July	29
Third harvest	19 August	30 September	44	6 July	7 August	34
Fourth harvest				8 August	September 20	44

a Start at the alfalfa seeding.

The dried samples were crushed into a fine powder and then sieved through a 0.5-mm mesh before the quality content characteristics, including acid detergent fiber, neutral detergent fiber, and crude protein, were assessed. Crude protein was evaluated using a FOSS Kjeltec™ 8400 (FOSS Ltd., Denmark) instrument, and acid detergent fiber and neutral detergent fiber were tested with an ANKOM2000 Automated Fiber Analyzer (ANKOM Technology, Macedon, NY, USA) in a bag suspender (Warnke and Ruhland, 2016).

The soil moisture was monitored every 2–3 days, utilizing 0.1 m depth increments within the vertical soil layer, extending to a depth of 1.6 m. This was achieved using a Diviner 2000 system (Sentek Pty Ltd., Australia). Calibration of the data was performed by comparing it with gravimetric soil water content, measured through the oven-drying method at each harvest stage. The soil water content reported here represents the average moisture level across all soil layers that were monitored.

### 2.3 Calculation

The relative feed value was estimated according to Equation 1.


(1) $$Relative\;feed\;value=\frac{120/Neutral\;detergent\;fiber\times (88.9-0.779\times Acid\;detergent\;fiber)}{1.29}$$

Alfalfa average acid detergent fiber yield, average neutral detergent fiber yield, average crude protein yield, and average relative feed value yield (Zhang et al., 2018; Deng et al., 2020) were calculated as **Equations 2**–**5**:


(2) Acid detergent fiber yield=Average hay yield×Acid detergent fiber (%)

(3) Neutral detergent fiber yield=Average hay yield×Neutral detergent fiber (%)

(4) Crude protein yield=Average hay yield×Crude protein (%)

(5) Relative feed value yield=Average hay yield×Relative feed value

### 2.4 Statistics

**Figure 3:**
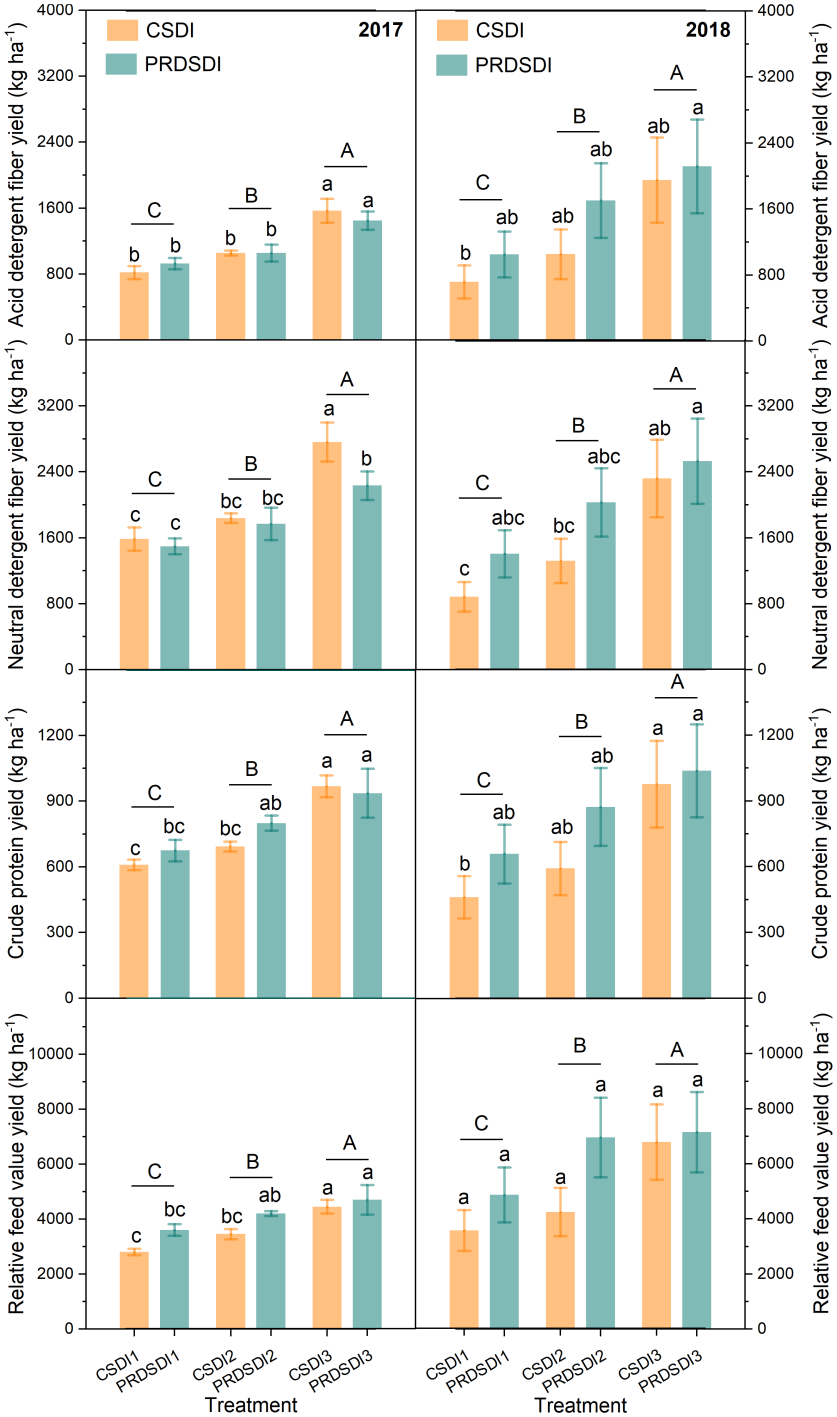
Alfalfa average quality yield at three harvests in 2017 and four harvests in 2018. CSDI represents conventional subsurface drip irrigation; PRDSDI represents partial root-zone drying subsurface drip irrigation. CSDI1 and PRDSDI1, CSDI2 and PRDSDI2, and CSDI3 and PRDSDI3 represent 10-mm, 20-mm, and 30-mm irrigation volumes at each irrigation event per week, respectively. Different lowercase letters indicate that the results of single factor analysis have significant differences. Different uppercase letters indicate that the results of different irrigation quotas have significant differences. (For interpretation of the references to color in this figure legend, the reader is referred to the web version of this article).

**Figure 4:**
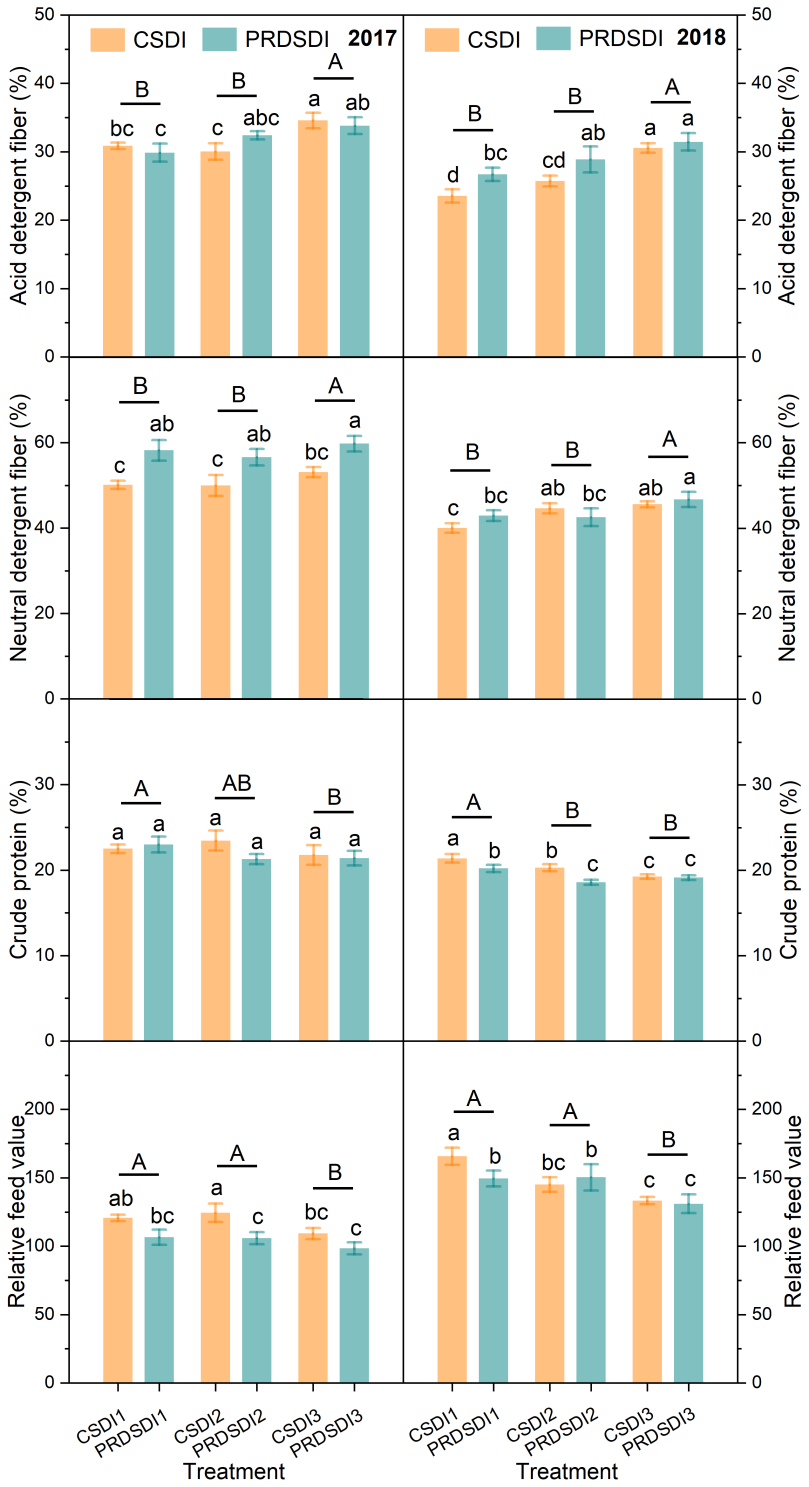
Alfalfa average quality content at three harvests in 2017 and four harvests in 2018. CSDI represents conventional subsurface drip irrigation; PRDSDI represents partial root-zone drying subsurface drip irrigation. CSDI1 and PRDSDI1, CSDI2 and PRDSDI2, and CSDI3 and PRDSDI3 represent 10-mm, 20-mm, and 30-mm irrigation volumes at each irrigation event per week, respectively. Different lowercase letters indicate that the results of single factor analysis have significant differences. Different uppercase letters indicate that the results of different irrigation volumes have significant differences. (For interpretation of the references to color in this figure legend, the reader is referred to the web version of this article).

**Figure 5:**
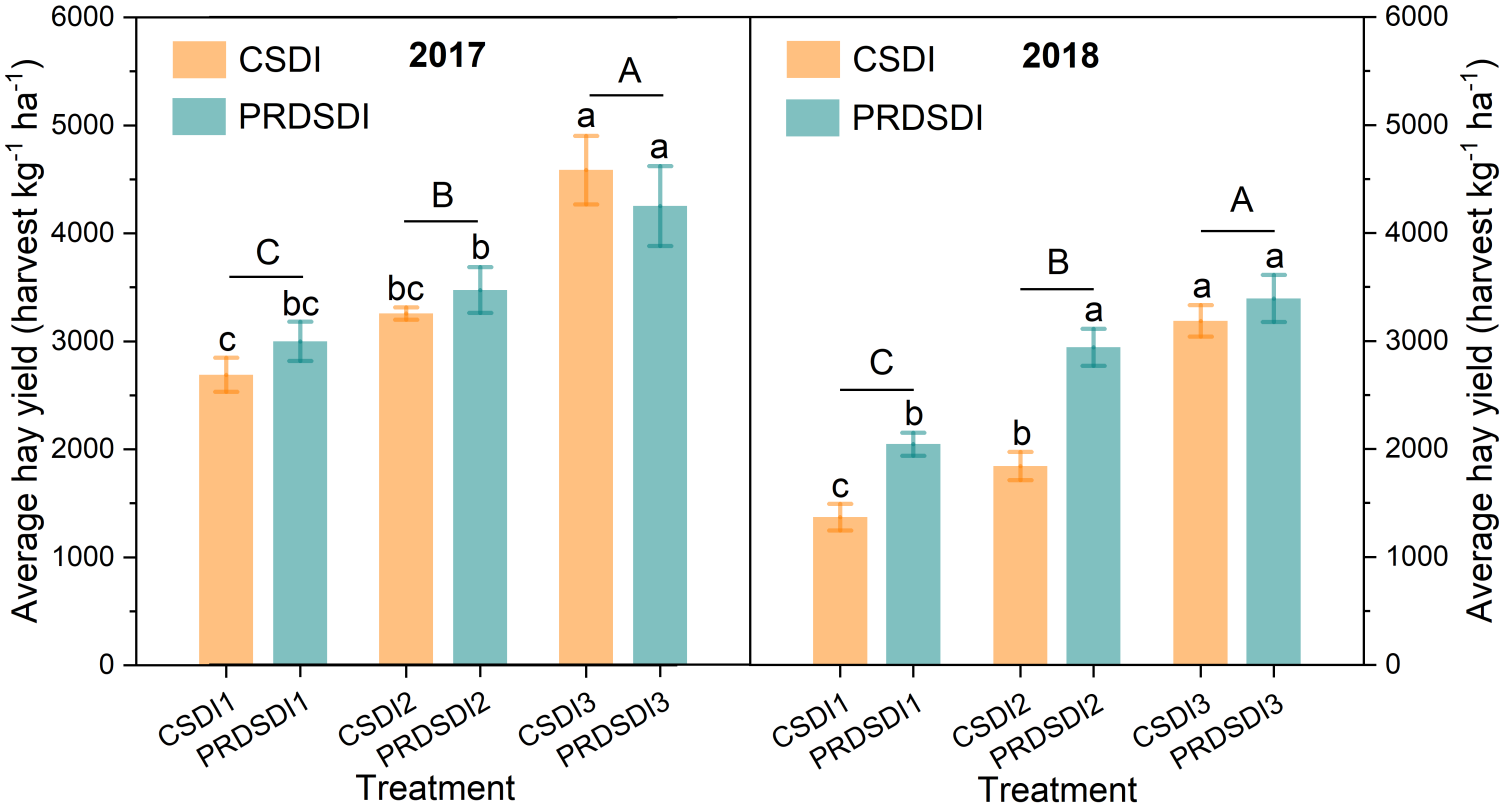
Alfalfa average hay yield at three harvests in 2017 and four harvests in 2018. CSDI represents conventional subsurface drip irrigation; PRDSDI represents partial root-zone drying subsurface drip irrigation. CSDI1 and PRDSDI1, CSDI2 and PRDSDI2, and CSDI3 and PRDSDI3 represent 10-mm, 20-mm, and 30-mm irrigation volumes at each irrigation event per week, respectively. Different lowercase letters indicate that the results of single factor analysis have significant differences. Different uppercase letters indicate that the results of different irrigation volumes have significant differences. (For interpretation of the references to color in this figure legend, the reader is referred to the web version of this article).

Analyses were performed using the SPSS 22 software (SPSS Inc., Chicago, IL, USA). Three-way analyses of variance (ANOVA) with the general linear model univariate procedure were used to test the effects of the harvest events (harvest event), irrigation methods, and irrigation volumes as well as their interaction on the alfalfa quality content (acid detergent fiber, neutral detergent fiber, crude protein, and relative feed value), average hay yield, and quality yield (acid detergent fiber yield, neutral detergent fiber yield, crude protein yield, and relative feed value yield). Different lowercase letters indicate that the results of one-way ANOVA have significant differences, and different uppercase letters indicate that the results of different irrigation volumes have significant differences in **Figures 3**–**5**. Principal component analysis of alfalfa average hay yield and quality characteristics at seven harvests in two years was conducted using Origin 2023b (Origin Lab Corp., Northampton, USA).

## 3 Results

### 3.1 Average volume moisture content

**Figure 6:**
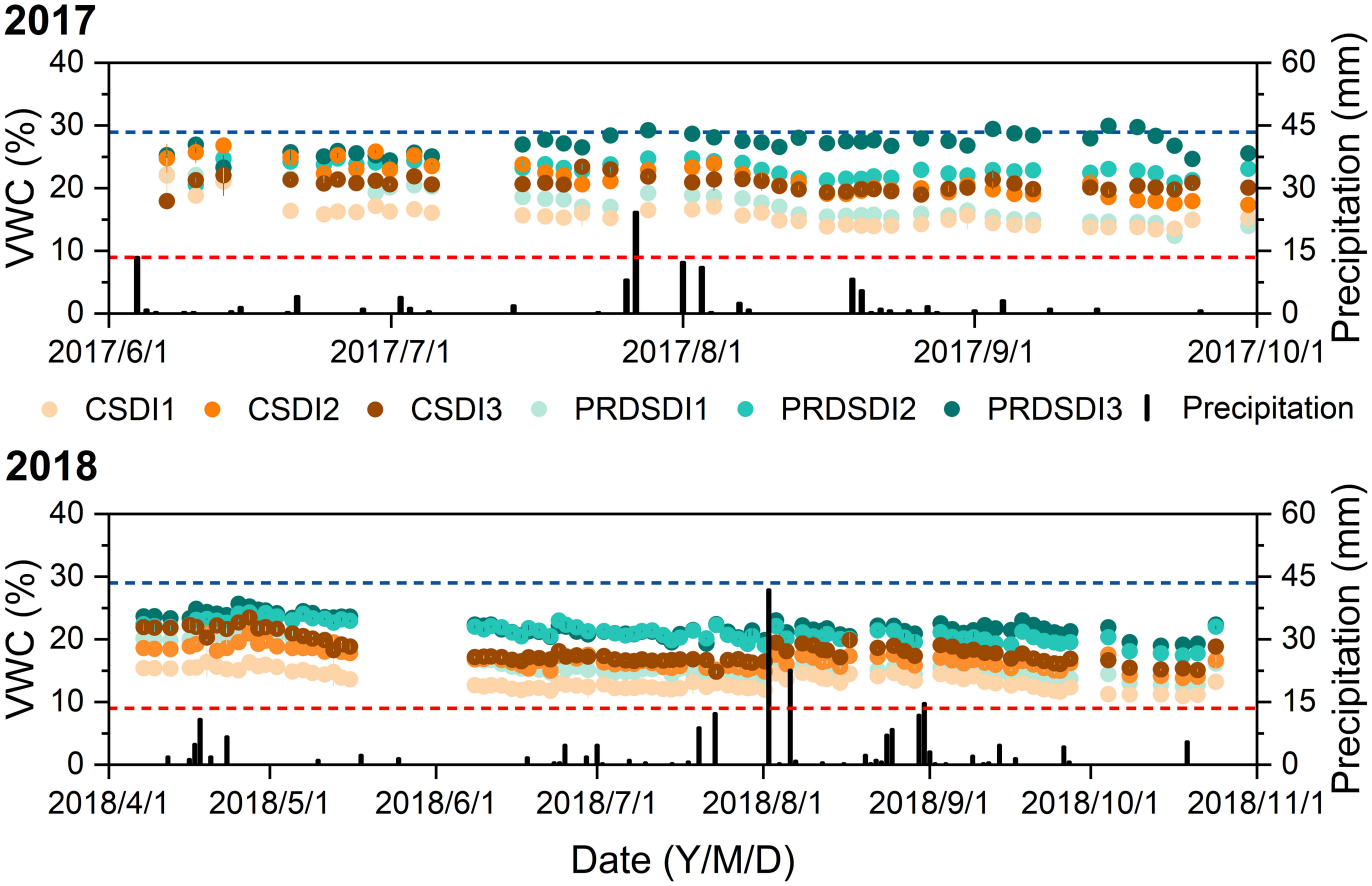
Average volume soil water content (VWC) dynamics of 10 cm to 160 cm under two irrigation methods (CSDI represents conventional subsurface drip irrigation; PRDSDI represents partial root-zone drying subsurface drip irrigation) and three irrigation volumes (CSDI1 and PRDSDI1, CSDI2 and PRDSDI2, and CSDI3 and PRDSDI3 represent 10-mm, 20-mm, and 30-mm irrigation volumes at each irrigation event per week, respectively) in 2017 and 2018. The blue line represents the field capacity, and the red line represents permanent wilting point, respectively. (For interpretation of the references to color in this figure legend, the reader is referred to the web version of this article).

The average volume moisture content was higher under PRDSDI than that under CSDI treatments, and PRDSDI3 exhibited the highest average volume moisture content among these treatments in both years (**Figure 6**). However, the average volume moisture content of PRDSDI3 reached the field capacity after the first harvest until the third harvest in 2017 (**Figure 6**).

### 3.2 Alfalfa quality yield

Irrigation method significantly affected alfalfa neutral detergent fiber yield and crude protein yield in both years (*P*< 0.01) (**Table 3**). In 2017, the irrigation method showed no significant impact on alfalfa acid detergent fiber yield and relative feed value yield (*P* > 0.05). However, in 2018, the irrigation method significantly affected alfalfa acid detergent fiber yield and relative feed value yield (*P*< 0.001). The interaction between the irrigation method and the irrigation volume significantly affected alfalfa acid detergent fiber yield, neutral detergent fiber yield, crude protein yield, and relative feed value yield in 2017 (*P*< 0.001). In 2018, the interaction between the irrigation method and the irrigation volume significantly affected alfalfa neutral detergent fiber yield, crude protein yield, and relative feed value yield (*P*< 0.05), whereas the interaction between the irrigation method and the irrigation volume did not significantly affect alfalfa acid detergent fiber yield.

**Table 3:** Three-way ANOVAs (F- and P-values) of the effects of the irrigation method, the irrigation volume, and the harvest event on the alfalfa quality content, hay yield, and quality yield in 2017 and 2018.

Years	Measurement indexes		Irrigation method	Irrigation volume	Harvest event	Irrigation method × irrigation volume	Irrigation method × harvest event	Irrigation volume × harvest event	Irrigation method × irrigation volume × harvest event
2017	Quality content	Acid detergent fiber	9.63**.	13.33***	18.77***	2.09ns.	0.07ns.	6.56***	3.94**
		Neutral detergent fiber	0.58ns.	0.34ns.	7.11**	35.71***	2.01ns.	17.38***	2.16ns.
		Crude protein	0.01ns	7.32**	44.45***	4.37*	4.44*	7.63***	3.82*
		Relative feed value	0.02ns.	4.30*	9.56***	0.94ns.	1.88ns.	15.87***	3.60*
		Hay yield	1.89ns.	448.25***	330.58***	21.03***	31.00***	44.02***	6.18***
	Quality yield	Acid detergent fiber yield	0.41ns	146.23***	111.96***	4.35*	8.87***	3.13*	3.39*
		Neutral detergent fiber yield	21.46***	159.34***	142.06***	34.08***	19.90***	4.01**	6.20**
		Crude protein yield	5.84*	176.54***	139.07***	6.83**	11.72***	51.64***	2.64*
		Relative feed value yield	0.03ns.	67.04***	44.09***	13.06***	3.17ns.	30.00***	1.16ns.
2018	Quality content	Acid detergent fiber	36.35***	18.39***	31.46***	30.23***	13.50***	2.50*	11.50***
		Neutral detergent fiber	40.49***	0.41ns.	19.65***	6.23**	6.60***	2.34*	18.24***
		Crude protein	13.95***	44.08***	64.47***	41.85***	2.56ns.	4.89***	8.24***
		Relative feed value	41.58***	1.91ns.	19.68***	9.22***	7.02***	1.35ns.	13.77***
		Hay yield	129.09***	247.92***	66.40***	19.57***	3.31*	4.86**	3.25**
	Quality yield	Acid detergent fiber yield	22.85***	158.36***	61.48***	2.68ns.	6.41***	6.45***	5.54***
		Neutral detergent fiber yield	39.03***	153.21***	63.95***	7.04**	4.05*	5.67***	5.82***
		Crude protein yield	139.98***	177.74***	34.55***	36.75***	2.13ns.	4.98***	5.60***
		Relative feed value yield	168.52***	129.12***	14.68***	23.65***	7.12***	2.97*	8.65***

*indicates significant differences at P = 0.05, **indicates significant differences at P = 0.01, ***indicates significant differences at P = 0.001, and ns. indicates not significant.

CSDI increased the neutral detergent fiber yield by 3.5% to 18.9% in 2017 compared with PRDSDI. Notably, CSDI3 significantly elevated neutral detergent fiber yield by 522.72 kg ha−^1^ compared with PRDSDI3 in 2017. There was no difference found in acid detergent fiber yield, neutral detergent fiber yield, crude protein yield, and relative feed value yield between the PRDSDI and CSDI in 2017 or 2018 using ANOVA (**Figure 3**). In 2017, compared with CSDI1, PRDSDI1 improved the acid detergent fiber yield, crude protein yield, and relative feed value yield by 13.6%, 11.0%, and 28.3%, respectively. Similarly, compared with CSDI2, PRDSDI1 improved the acid detergent fiber yield, crude protein yield, and relative feed value yield by 0.2%, 15.6%, and 21.8% in 2017, respectively. In 2018, compared with CSDI, PRDSDI improved the acid detergent fiber yield, neutral detergent fiber yield, crude protein yield, and relative feed value yield by 9.0%–59.1%, 9.1%–63.7%, 6.1%–47.0%, and 5.1%–63.8%, respectively.

### 3.3 Alfalfa quality content

Irrigation method significantly influenced alfalfa acid detergent fiber and crude protein (*P<* 0.001), but no significant effects were observed on neutral detergent fiber and relative feed value in 2017 (**Table 3**). In 2018, the irrigation method had a significant impact on alfalfa neutral detergent fiber and relative feed value (*P<* 0.001), whereas it did not significantly affect acid detergent fiber and crude protein. The interaction between the irrigation method and the irrigation volume had a significant effect on alfalfa acid detergent fiber and crude protein (*P<* 0.05), whereas it did not significantly affect neutral detergent fiber and relative feed value in 2017. In 2018, the interaction between the irrigation method and the irrigation volume had a significant effect on alfalfa neutral detergent fiber, crude protein, and relative feed value (*P<* 0.05), whereas it did not significantly affect acid detergent fiber.

ANOVA analysis indicated that PRDSDI significantly increased the alfalfa acid detergent fiber and neutral detergent fiber and significantly decreased crude protein and relative feed value compared with CSDI in 2017 and 2018 (*P<* 0.05) (**Figure 4**). Specifically, alfalfa neutral detergent fiber in 2017 and acid detergent fiber in 2018 of PRDSDI1 were higher by 16.2% and 13.3% than that of CSDI1, respectively. In addition, alfalfa crude protein and relative feed value of PRDSDI1 were lower by 5.4% and 9.8% than those of CSDI1 in 2018. The alfalfa neutral detergent fiber in 2017 and acid detergent fiber in 2018 of PRDSDI2 were higher by 13.2% and 12.2% than that of CSDI2, respectively. Conversely, alfalfa crude protein in 2018 and relative feed value in 2017 of PRDSDI2 were lower by 8.3% and 15.0% than that of CSDI2 in 2018. In addition, the alfalfa neutral detergent fiber of PRDSDI3 was higher by 12.6% than that of CSDI3 in 2017.

### 3.4 Alfalfa average hay yield

The irrigation method insignificantly affected alfalfa average hay yield in 2017 (*P* = 0.178), but both the irrigation method and the irrigation volume, along with their interaction, significantly influenced alfalfa average hay yield in 2017 and 2018 (*P<* 0.001) (**Table 3**). The average hay yield of PRDSDI1 and PRDSDI2 was slightly higher by 304.96 and 212.84 harvest kg^−1^ ha^−1^ than that of CSDI1 and CSDI2 in 2017 (*P* > 0.05). However, in 2018, the alfalfa average hay yield of PRDSDI1 and PRDSDI2 was significantly higher by 1,083.52 and 1,760.48 harvest kg^−1^ ha^−1^ than that of CSDI1 and CSDI2, respectively (**Figure 5**).

### 3.5 Principal component analysis of the alfalfa quality characteristics and hay yield

**Figure 7:**
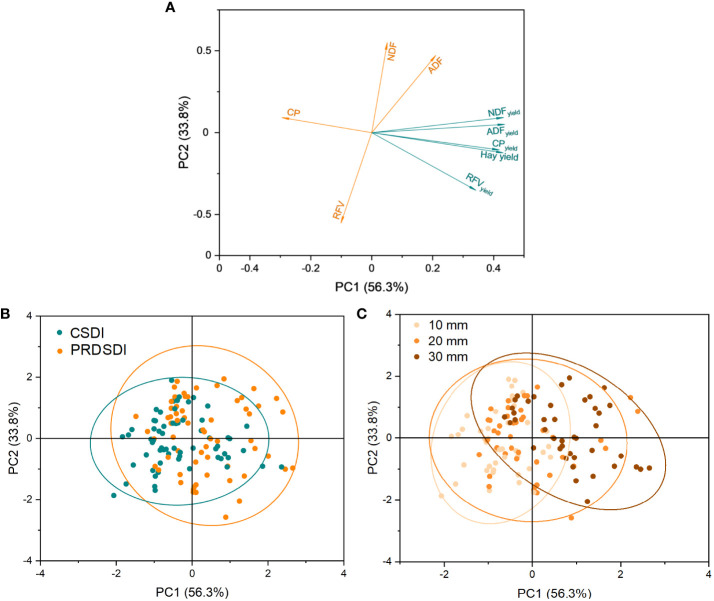
Two-dimensional representation of the areas defined by the first two principal components (PC1 and PC2) of the principal component analysis of the alfalfa quality characteristics **(A)** between CSDI and PRDSDI **(B)** and among three irrigation volumes **(C)** at seven harvests in 2017–2018. Values assigned to each alfalfa sample for PC1 and PC2 grouped by irrigation method **(B)** and irrigation volumes **(C)**. CSDI represents conventional subsurface drip irrigation; PRDSDI represents partial root-zone drying subsurface drip irrigation; 10 mm, 20 mm, and 30 mm represent irrigation volumes at each irrigation event per week. The relative contribution of each variable to PC1 and PC2. CP, crude protein; ADF, acid detergent fiber; NDF, neutral detergent fiber; RFV, relative feed value; ADF_yield_, acid detergent fiber yield; NDF_yield_, neutral detergent fiber yield; CP_yield_, crude protein yield; RFV_yield_, relative feed value yield. The orange circle represents the confidence interval under PRDSDI, and the green circle represents the confidence interval under CSDI (for interpretation of the references to color in this figure legend, the reader is referred to the web version of this article.).

The principal component analysis conducted on the nine most important quality characteristics captured 90.1% of the variance in the first two axes with 56.3% on the first component (PC1) at seven harvests in 2017 and 2018 (**Figure 7**). PC1 exhibited a negative correlation with alfalfa relative feed value and crude protein, whereas it showed a positive correlation with alfalfa acid detergent fiber, neutral detergent fiber, hay yield, and quality yield (acid detergent fiber yield, neutral detergent fiber yield, crude protein yield, and relative feed value yield) (**Figure 7A**). With respect to irrigation methods, the principal component analysis scores indicated that PC1 discriminate between CSDI and PRDSDI (**Figure 7B**). CSDI was characterized by a higher alfalfa relative feed value and crude protein, whereas PRDSDI was characterized by a higher alfalfa hay yield and quality yield. The second principal component (PC2) explained 33.8% of the variation, showing a negative relationship with relative feed value and relative feed value yield but a positive relationship with acid detergent fiber and neutral detergent fiber. PC2 did not discriminate between PRDSDI and CSDI. The PRDSDI was at the positive end of PC2 and had high acid detergent fiber and neutral detergent fiber. PRDSDI was found mostly on the negative side of PC2, with a higher relative feed value than CSDI. Furthermore, principal component analysis based on the irrigation volume (**Figure 7C**) demonstrated a positive association between the higher irrigation volume and alfalfa hay yield and quality yield while revealing a negative correlation with quality content.

## 4 Discussion

In this study, the alfalfa quality yield, quality content, and average hay yield under two irrigation methods and three irrigation volumes were investigated in a 2-year-old field experiment. Our key point is the changes in the alfalfa quality yield, quality content, and average hay yield with irrigation methods and interaction with irrigation volumes. The changes in the alfalfa quality yield were explained from the aspects of quality content and average hay yield.

### 4.1 PRDSDI has the potential to improve the alfalfa quality yield

**Figure 7**, **Table 3**, and **Supplementary Figure 1** collectively highlight the potential of PRDSDI in enhancing the alfalfa quality yield, aligning with our initial hypothesis. To our knowledge, this study represents the first exploration of the alfalfa quality yield under PRDSDI. The interaction between irrigation methods and irrigation volumes in both years significantly affected the alfalfa quality yield, indicating that the effects of irrigation methods on the alfalfa quality cannot be ignored and depend on the deficit irrigation degree of irrigation volumes. Previous studies have reported that reduced irrigation volume significantly decreases the crude protein yield of soybean, oat, and vetch (Lai et al., 2022), and their results also support deficit irrigation cut-down forage quality yield. This is also consistent with our results, where 1/3–2/3 irrigation volumes of full irrigation significantly reduced the alfalfa quality yield (**Figure 3**). **Figure 7**, **Table 3**, and **Supplementary Figure 1** collectively highlight the potential of PRDSDI in enhancing the alfalfa quality yield, aligning with our initial hypothesis. To our knowledge, this study represents the first exploration of the alfalfa quality yield under PRDSDI. The interaction between irrigation methods and irrigation volumes in both years significantly affected the alfalfa quality yield, indicating that the effects of irrigation methods on the alfalfa quality cannot be ignored and depend on the deficit irrigation degree of irrigation volumes. Previous studies have reported that reduced irrigation volume significantly decreases the crude protein yield of soybean, oat, and vetch (Lai et al., 2022), and their results also support deficit irrigation cut-down forage quality yield. This is also consistent with our results, where 1/3–2/3 irrigation volumes of full irrigation significantly reduced the alfalfa quality yield (**Figure 3**). When deficit irrigation occurs, PRDSDI improved the alfalfa acid detergent fiber yield, neutral detergent fiber yield, and crude protein yield in two years (**Figures 3**, **7**) and reversed the negative effects on acid detergent fiber yield and neutral detergent fiber yield of PRDSDI in 2017 (**Figure 3**). These results suggest that optimizing the combination of irrigation methods and irrigation volumes is crucial for improving the alfalfa quality as a forage crop. Our results provide novel insights into the effects of irrigation methods and irrigation volumes on alfalfa quality. These results underscore the pivotal role of optimizing the combination of irrigation methods and irrigation volumes in enhancing the alfalfa quality as a forage crop, providing valuable insights for farm management and policy decisions.

Notably, we observed that the accumulation of crude protein yield and relative feeding value yield of alfalfa treated with PRDSDI2 in 2018 was not inferior to the highest irrigation volume (PRDSDI3 and CSDI3) (**Supplementary Figure 1**). The introduced indicator of the alfalfa quality yield effectively evaluates the trade-off between quality content and hay yield under PRDSDI and CSDI, serving as a practical tool for informed decision-making at both the farm and policy levels. This indicator has found utility in forage field management and other natural grassland ecosystems (Holman et al., 2016; Wang et al., 2020; Kamran et al., 2022), as well as in other natural grassland ecosystems (Wang et al., 2011; Grant et al., 2014; Schaub et al., 2020; Li et al., 2021).

### 4.2 PRDSDI decreased the alfalfa quality content

One-way ANOVA and principal component analysis demonstrated that PRDSDI increased alfalfa acid detergent fiber, neutral detergent fiber, reduced alfalfa crude protein, and relative feed value compared with CSDI (**Figures 4**, **7**). This unexpected outcome contradicts our initial hypothesis and stands out as one of the few studies reporting a negative impact of PRDSDI on plant quality. Previous research has consistently shown positive effects of PRDSDI on the quality content of vegetables and fruits under deficit irrigation compared with CSDI (O'Connell and Goodwin, 2007a; O'Connell and Goodwin, 2007b; Jovanovic et al., 2010; Çolak et al., 2018). However, it is worth noting that these studies typically focused on tubers or fruit, whereas our investigation centered around alfalfa biomass harvesting (Xiao et al., 2015; Zhang et al., 2020; Zhang et al., 2021). Zhang and colleagues (2021) found that partial root-zone drying based on furrow irrigation could significantly increase nitrogen content in alfalfa stems and leaves, which indicated that partial root-zone drying under furrow irrigation could improve the alfalfa quality content, contrary to our results. This prompts speculation that the discrepancy in quality content may arise from the use of different fundamental irrigation methods.

The only study attempting to elucidate the mechanism of quality content based on a pot experiment suggested that PRDSDI increased metabolite content in potato tubers by minimizing the decrease in glucose and fructose concentrations and doubling the amount of mannitol compared with CSDI (Elhani et al., 2019). Although PRDSDI has been observed to increase wheat proline (a protein component) (Raza et al., 2017), the negative mechanism of PRDSDI on the alfalfa quality content remains poorly understood, necessitating further in-depth exploration.

Wang et al. (2021) identified significant uncertainty in the impact of irrigation methods on the alfalfa quality content under full irrigation. In their study, PRDSDI significantly reduced the acid detergent fiber of 1-year-old alfalfa at the third harvest compared with CSDI, thereby improving overall quality content under full irrigation (Wang et al., 2021). However, under full irrigation, PRDSDI increased the acid detergent fiber and neutral detergent fiber of 2-year-old alfalfa at the second harvest, whereas it reduced the neutral detergent fiber at the fourth harvest, resulting in decreased the overall alfalfa quality content (Wang et al., 2021). Our observations revealed slight fluctuations in PRDSDI with acid detergent fiber with 10-mm and 30-mm irrigation volumes in 2017 and neutral detergent fiber with a 20-mm irrigation volume in 2018. Although no significant differences were observed between PRDSDI and CSDI in these instances (**Figure 4**), uncertainties in alfalfa neutral detergent fiber and relative feed value were still present at the fourth harvest in 2018 (**Supplementary Figures 3**, **5**). Consequently, the response of the alfalfa quality content to PRDSDI appears to be complex, warranting further research to comprehensively compare the effects of PRDSDI on the alfalfa quality content.

The negative correlation between irrigation volume and quality content (**Figures 7A, C**) implies that reducing irrigation volumes intensifies interactions with irrigation methods (**Table 3**). This suggests that the impact of incorporating deficit irrigation implementations tends to be amplified when we consider the effects of irrigation volumes. Some research partial root-zone drying is usually similar irrigation volume to deficit irrigation; thus, it is difficult to distinguish the influence of irrigation methods and irrigation volumes on the quality effect (Tang et al., 2005; Jovanovic et al., 2010; Romero et al., 2016). A pot experiment on partial root-zone drying research also found the interaction between the irrigation method and the irrigation volume in potato antioxidant activity, total polyphenols, and sugars (Elhani et al., 2019). Consequently, conducting more interactive experiments to explore the effect of partial root-zone drying with optimal deficit irrigation gradients for the best quality content is recommended.

### 4.3 PRDSDI increased the alfalfa hay yield under deficit irrigation

In contrast to the alfalfa quality content, our findings indicate that PRDSDI enhanced the average hay yield of seven harvests with 1/3–2/3 irrigation volumes of full irrigation in 2018 compared with CSDI (**Figure 5**). The positive effects of PRDSDI on yields align with observations in other crops (O'Connell and Goodwin, 2007a; O'Connell and Goodwin, 2007b; Jovanovic et al., 2010; Çolak et al., 2018; Liu et al., 2021b). It is worth noting that PRDSDI did not exhibit a significant yield-enhancing effect under full irrigation in the previous study (Wang et al., 2021). In their research, PRDSDI with full irrigation actually led to a reduction in annual 1-year-old alfalfa hay yield, with no significant effect on 2-year-old alfalfa hay yield compared with CSDI. Our results complement this research by demonstrating that PRDSDI can mitigate average hay yield losses under moderate deficit irrigation (2/3 irrigation volume of full irrigation), achieving average hay yields that are not significantly different from full irrigation (**Figure 3**). Given that deficit irrigation is a common practice in the Northwest of China and other water-limited regions, our findings suggest that PRDSDI provides a promising irrigation method to enhance alfalfa production in such areas.

In addition, we observed consistent responses in the 2-year average hay yield to both PRDSDI and CSDI under deficit irrigation (**Figure 5**). In 2017, there was a slight improvement in PRDSDI for average hay yield under deficit irrigation compared with that in CSDI, although no statistically significant differences were detected. We recommend that 1-year-old alfalfa should not undergo deficit irrigation, as it poses a risk of average hay yield loss. Notably, average hay yields’ significant improvement with PRDSDI under deficit irrigation compared with CSDI was evident in 2018, approaching no significant difference under full irrigation conditions (**Figure 5**). Thus, employing novel PRDSDI in 2-year-old alfalfa under moderate deficit irrigation conditions ensures that hay yield is not less than that under full irrigation.

We also observed a potential reduction in hay production in 2018 for CSDI under deficit irrigation (CSDI1 and CSDI2), a trend also noted for PRDSDI1. This phenomenon may be attributed to maintaining the same irrigation treatment in 2018 as in 2017 within the same plot, potentially leaving a lasting impact of the water deficit treatment on alfalfa yield from 2017 to 2018. This suggests the occurrence of drought legacy effects (De Boeck et al., 2018; Jing et al., 2022). Further research is warranted to unravel the underlying mechanisms of these observations.

## 5 Conclusion

The 2 years of field experiments aimed to investigate the impact of PRDSDI and CSDI on the alfalfa quality yield, quality content, and average hay yield. The results demonstrated that, in comparison with CSDI, PRDSDI led to an improvement in the alfalfa quality yield. This improvement was elucidated by the increase in average hay yield as the quality content decreased. The interaction between the irrigation method and the irrigation volume significantly impacted the alfalfa quality yield, quality content, and average hay yield in the majority of cases. These findings underscore the necessity of considering the trade-off at irrigation volume gradients between the alfalfa quality content and the average hay yield when employing PRDSDI and CSDI in water management. We recommend that alfalfa can be fully irrigated with PRDSDI. Furthermore, moderate deficit irrigation of 2-year-old alfalfa has the potential to increase neutral detergent fiber and relative feeding value without causing a significant reduction in hay yields. Subsequently, our forthcoming research will focus on conducting a profit analysis based on the alfalfa quality content and hay yield under these two irrigation methods and volumes, aiming to contribute to the maximization of profitability in forage production systems at both the farm and policy levels.

## Data availability statement

The original contributions presented in the study are included in the article/**Supplementary Material**. Further inquiries can be directed to the corresponding authors.

## Author contributions

YW: Investigation, Methodology, Writing – original draft, Writing – review & editing. CX: Conceptualization, Visualization, Writing – review & editing. YS: Formal analysis, Writing – review & editing. JC: Formal analysis, Writing – review & editing. HW: Writing – review & editing, Project administration, Resources, Writing – original draft. QG: Writing – review & editing. JH: Conceptualization, Writing – review & editing. XL: Visualization, Writing – review & editing. LH: Formal analysis, Visualization, Writing – review & editing. DS: Funding acquisition, Resources, Visualization, Writing – review & editing, Writing – original draft.
