# Supplementary material for: Partial root-zone drying subsurface drip irrigation increased the alfalfa quality yield but decreased the alfalfa quality content
Source: Front Plant Sci. 2024 Feb 6;15:1297468. doi: 10.3389/fpls.2024.1297468 (PMC10877020; doi:10.3389/fpls.2024.1297468)
Supplement: Supplementary file 1 [file DataSheet_1.docx]

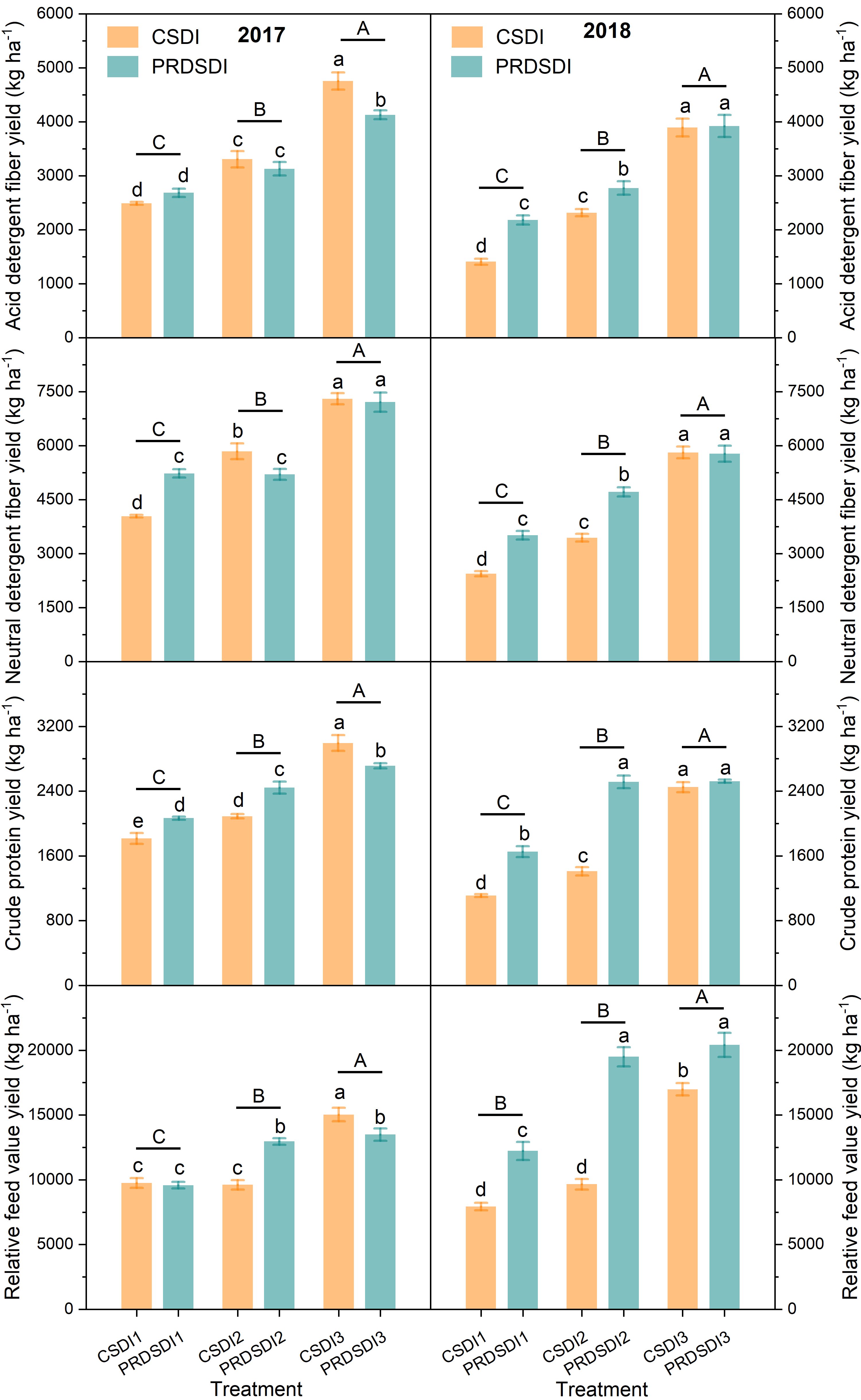


**Supplementary Figure 1.** Alfalfa quality yield in 2017 and 2018. PRDSDI represent partial root-zone drying subsurface drip irrigation; CSDI represent conventional subsurface drip irrigation. CSDI1 and PRDSDI1, CSDI2 and PRDSDI2, CSDI3 and PRDSDI3 represent 10 mm, 20 mm, and 30 mm irrigation volumes at each irrigation event per week, respectively. Different lowercase letters indicate that the results of single factor analysis have significant differences. Different uppercase letters indicate that the results of different irrigation quotas have significant differences. (For interpretation of the references to color in this figure legend, the reader is referred to the web version of this article).


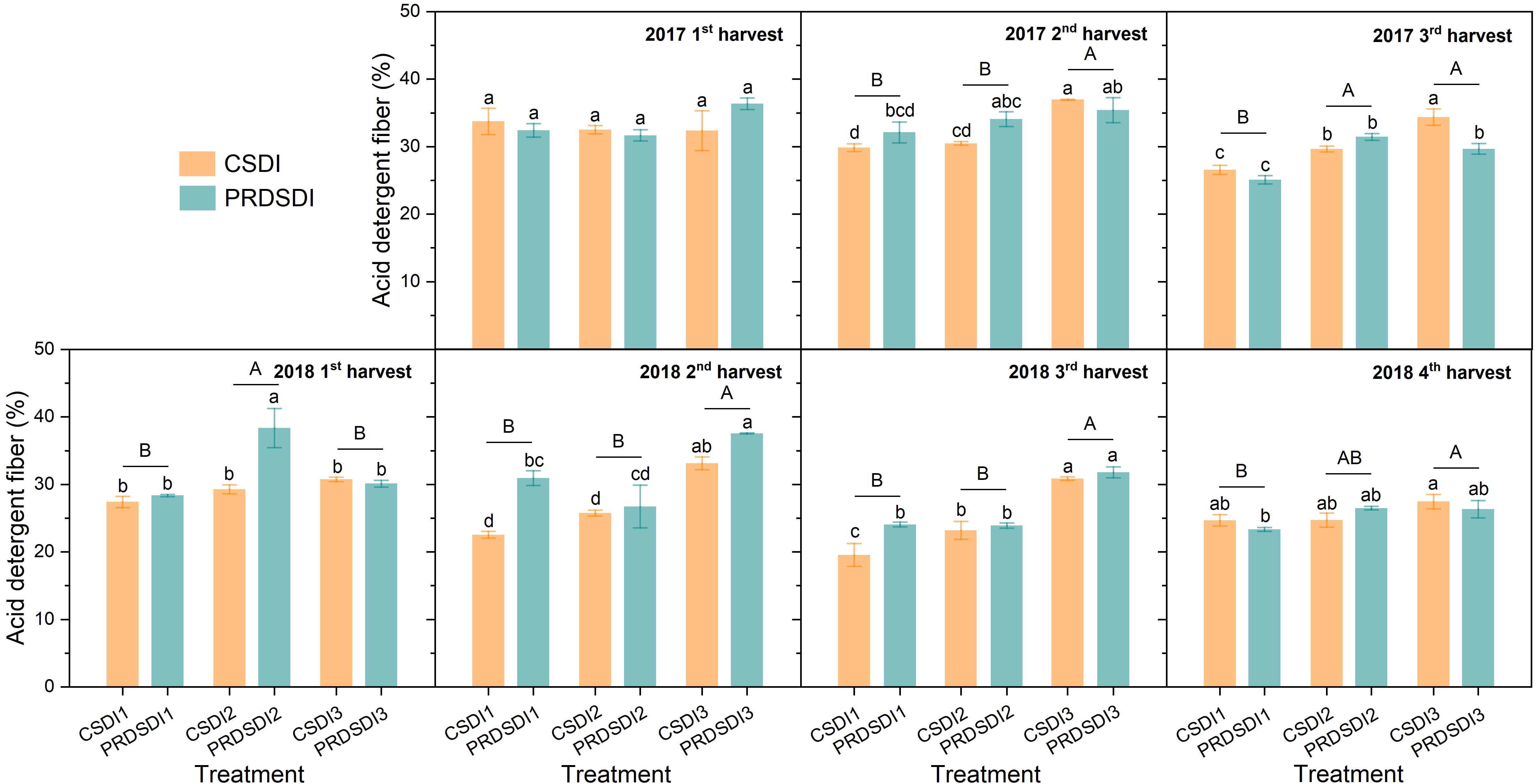


**Supplementary Figure 2.** Alfalfa acid detergent fiber at three harvests in 2017 and four harvests in 2018. CSDI represents convention subsurface drip irrigation; PRDSDI represents partial root-zone drying subsurface drip irrigation. CSDI1 and PRDSDI1, CSDI2 and PRDSDI2, CSDI3 and PRDSDI3 represent 10, 20, and 30 mm irrigation volumes at each irrigation event respectively. Different lowercase letters indicate that the results of single factor analysis have significant differences. Different uppercase letters indicate that the results of different irrigation quotas have significant differences. (For interpretation of the references to color in this figure legend, the reader is referred to the web version of this article).


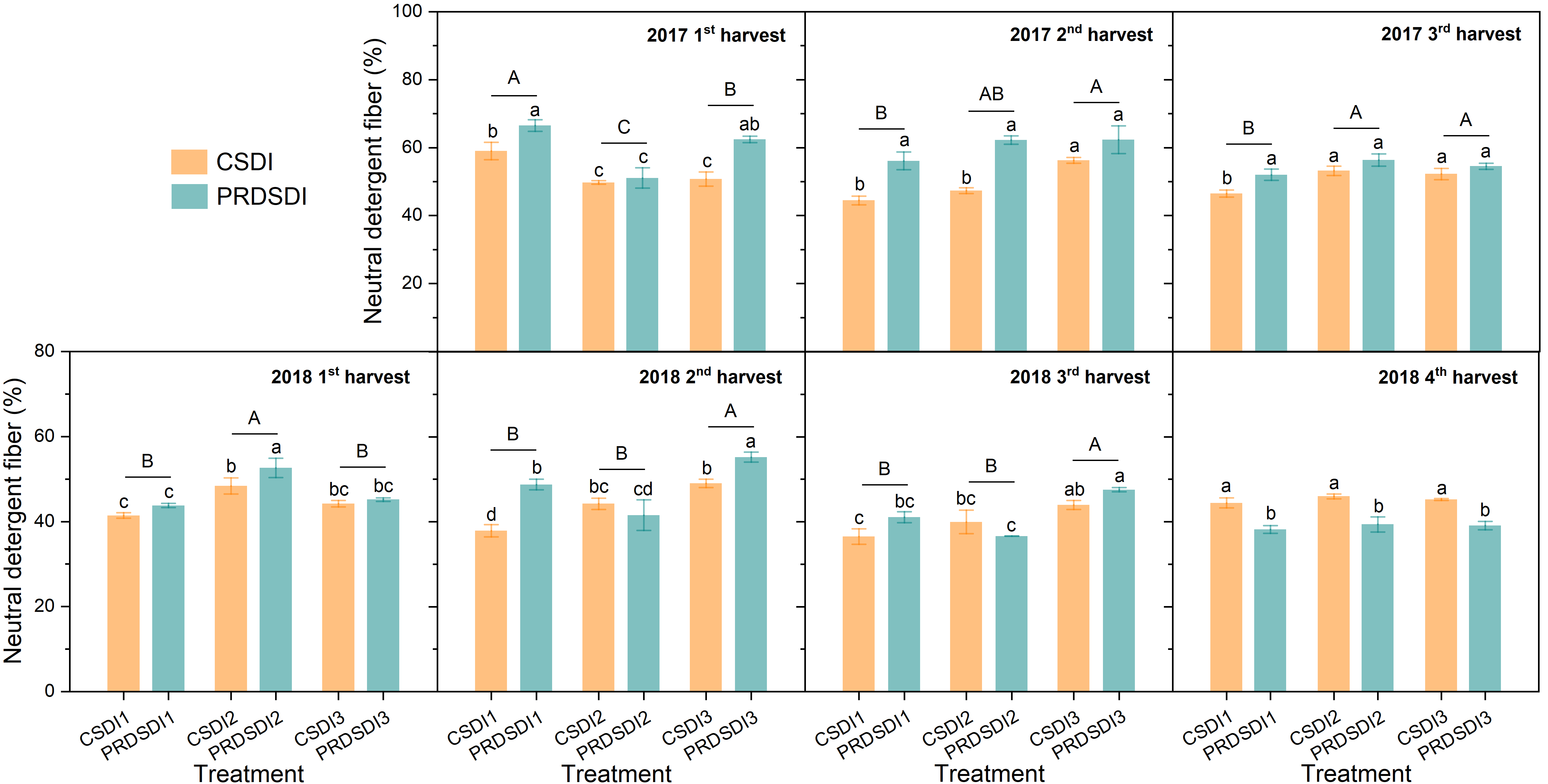


**Supplementary Figure 3.** Alfalfa neutral detergent fiber at three harvests in 2017 and four harvests in 2018. CSDI represents convention subsurface drip irrigation; PRDSDI represents partial root-zone drying subsurface drip irrigation. CSDI1 and PRDSDI1, CSDI2 and PRDSDI2, CSDI3 and PRDSDI3 represent 10, 20, and 30 mm irrigation volumes at each irrigation event respectively. Different lowercase letters indicate that the results of single factor analysis have significant differences. Different uppercase letters indicate that the results of different irrigation quotas have significant differences. (For interpretation of the references to color in this figure legend, the reader is referred to the web version of this article).


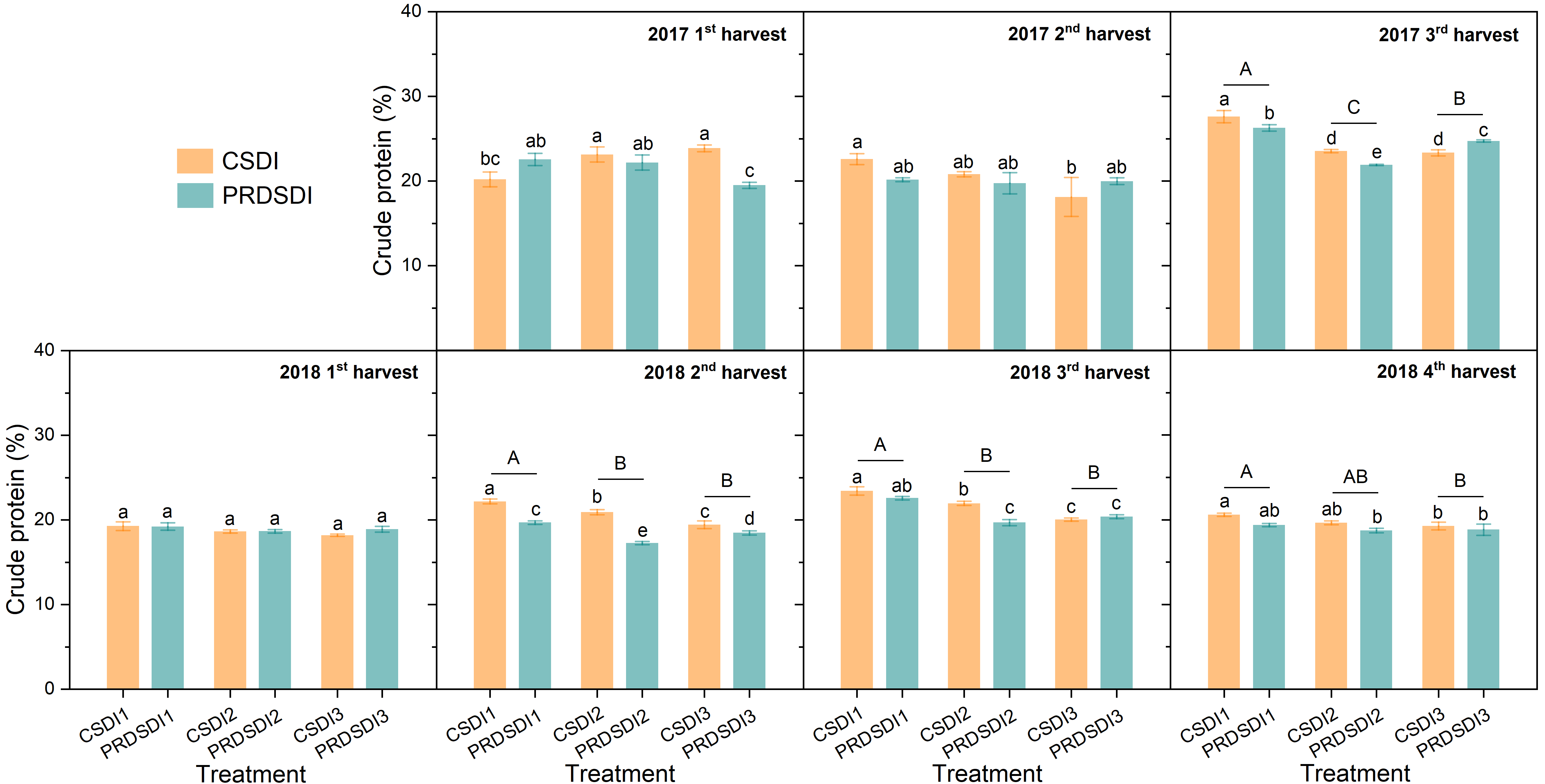


**Supplementary Figure 4.** Alfalfa crude protein at three harvests in 2017 and four harvests in 2018. CSDI represents convention subsurface drip irrigation; PRDSDI represents partial root-zone drying subsurface drip irrigation. CSDI1 and PRDSDI1, CSDI2 and PRDSDI2, CSDI3 and PRDSDI3 represent 10, 20, and 30 mm irrigation volumes at each irrigation event respectively. Different lowercase letters indicate that the results of single factor analysis have significant differences. Different uppercase letters indicate that the results of different irrigation quotas have significant differences. (For interpretation of the references to color in this figure legend, the reader is referred to the web version of this article).


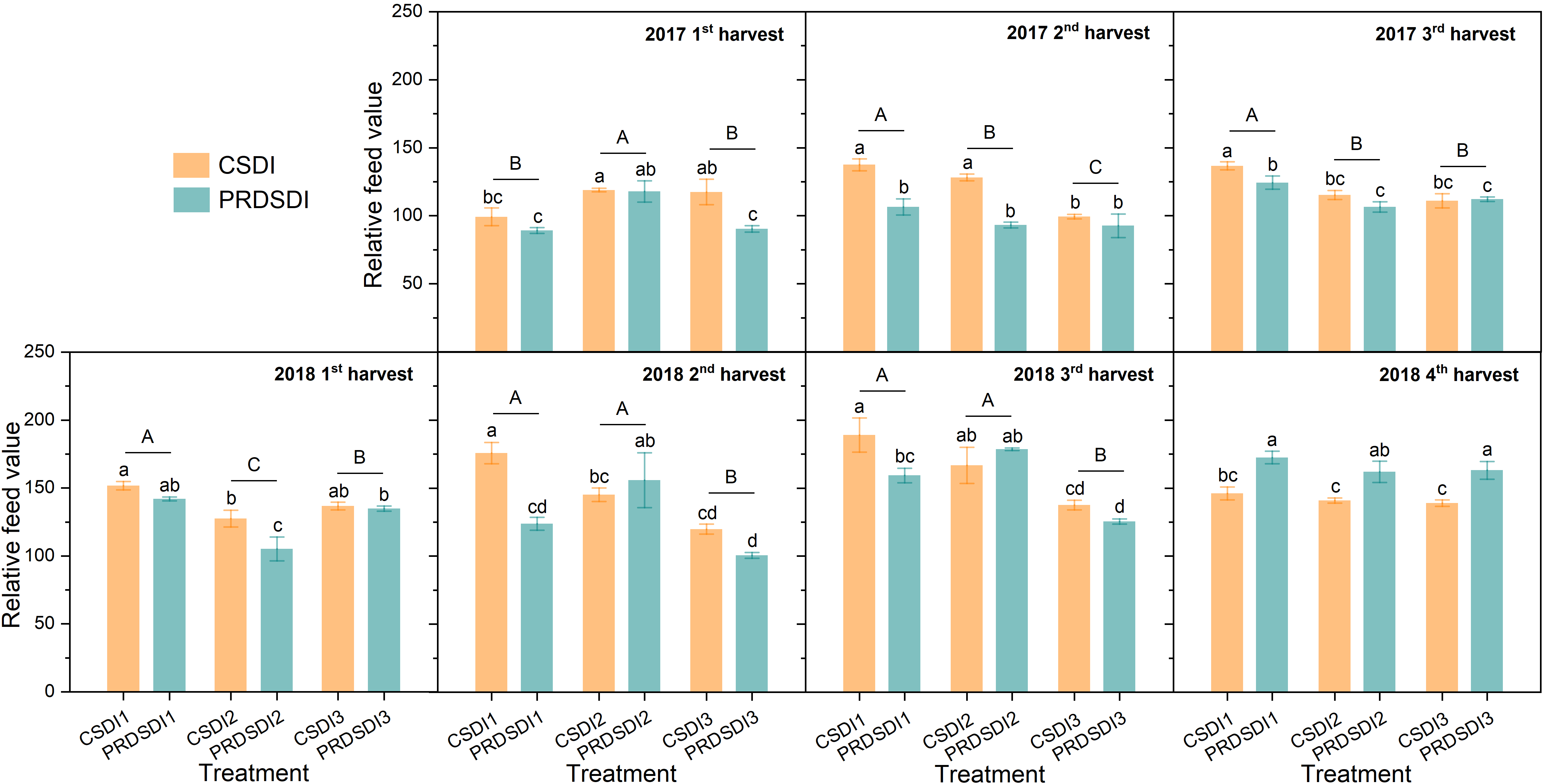


**Supplementary Figure 5.** Alfalfa relative feed value at three harvests in 2017 and four harvests in 2018. CSDI represents convention subsurface drip irrigation; PRDSDI represents partial root-zone drying subsurface drip irrigation. CSDI1 and PRDSDI1, CSDI2 and PRDSDI2, CSDI3 and PRDSDI3 represent 10, 20, and 30 mm irrigation volumes at each irrigation event respectively. Different lowercase letters indicate that the results of single factor analysis have significant differences. Different uppercase letters indicate that the results of different irrigation quotas have significant differences. (For interpretation of the references to color in this figure legend, the reader is referred to the web version of this article).
